# Molecular cytogenetic characterization and fusarium head blight resistance of five wheat-*Thinopyrum intermedium* partial amphiploids

**DOI:** 10.1186/s13039-021-00536-3

**Published:** 2021-03-06

**Authors:** Hui Wang, Shuwei Cheng, Yue Shi, Shuxin Zhang, Wei Yan, Weifu Song, Xuefeng Yang, Qingjie Song, Bo Jang, Xiaoyue Qi, Xinling Li, Bernd Friebe, Yanming Zhang

**Affiliations:** 1 Key Laboratory of Molecular Cytogenetics and Genetic Breeding of Heilongjiang Province, College of Life Science and Technology, Harbin Normal University, Harbin, 150025 China; 2 Crop Resources Institute, Heilongjiang Academy of Agriculture Sciences, Harbin, 150086 China; 3 Department of Plant Pathology, Wheat Genetics Resource Center, Throckmorton Plant Sciences Center, Kansas State University, Manhattan, KS 66506-5502 USA

**Keywords:** *Thinopyrum intermedium*, Common wheat, Partial amphiploids, Fusarium head blight, In situ hybridization

## Abstract

**Background:**

Partial amphiploids created by crossing octoploid tritelytrigia(2n = 8× = 56, AABBDDEE) and *Thinopyrum intermedium* (2n = 6× = 42, StStJJJ^S^J^S^) are important intermediates in wheat breeding because of their resistance to major wheat diseases. We examined the chromosome compositions of five wheat-*Th. intermedium* partial amphiploids using GISH and multicolor-FISH.

**Results:**

The result revealed that five lines had 10-14 J-genome chromosomes from *Th. intermedium* and 42 common wheat chromosomes, using the J-genomic DNA from *Th. bessarabicum* as GISH probe and the oligo probes *pAs1-1*, *pAs1-3*, *AFA-4*, (*GAA*) 10, and *pSc119.2-1* as FISH probe. Five lines resembled their parent octoploid tritelytrigia (2n = 8× = 56, AABBDDEE) but had higher protein contents. Protein contents of two lines HS2-2 and HS2-5 were up to more than 20%. Evaluation of Fusarium head blight (FHB) resistance revealed that the percent of symptomatic spikelets (PSS) of these lines were below 30%. Lines HS2-2, HS2-4, HS2-5, and HS2-16 were less than 20% of PPS. Line HS2-5 with 14 J-genome chromosomes from *Th. intermedium* showed the best disease resistance, with PSS values of 10.8% and 16.6% in 2016 and 2017, respectively.

**Conclusions:**

New wheat-*Th. intermedium* amphiploids with the J-genome chromosomes were identified and can be considered as a valuable source of FHB resistance in wheat breeding.

## Background

Common wheat (*Triticum aestivum* L, 2n = 6× = 42, AABBDD) is represented by a narrow germplasm base, which causes vulnerability to biotic and abiotic stresses [23, 35, 50, 55]. This narrow gene pool minimizes opportunities for developing genetic resistance to diseases. However, wild relatives of wheat provide a valuable reservoir of genes for cultivar improvement via wide hybridization [59, 69]. The wheatgrass, *Th. intermedium* (Host) Barkworth and D. R. Dewey (2n = 6× = 42) [syn. *Agropyron intermedium* (Host) Beauvoir, or *Elytrigia intermedia* (Host) Nevski, 2n = 6× = 42 StStJJJ^s^J^s^], is a perennial autoallo-hexaploid species that is an important source of genetic variability for improving cultivated wheat. It has been used extensively for hybridization with bread wheat and durum wheat, and numerous useful genes have been transferred to wheat [27, 51, 65]. Many derivatives have been produced from wheat-*Th. intermedium* hybrids, such as octoploid and hexaploid partial amphiploids and chromosome addition, substitution and translocation lines [10, 60]. *Th. intermedium* provides resistance against a wide spectrum of fungal pathogens (wheat leaf rust, stripe rust, stem rust, powdery mildew and eyespot; immunity to smut, leaf blight, root rot) and barley yellow dwarf virus and stripe mosaic viruses [13, 17, 23, 40, 41, 67]. Additionally, *Th. intermedium* is one of the most advanced examples of a recently domesticated perennial grain crop [18]. At present, numerous intergeneric hybrids and cytogenetic stocks have been developed from wheat-*Th. intermidium* crosses, including partial amphiploids [4, 22, 56], chromosome addition lines [29, 44], substitution lines [31, 38, 45], and translocation lines [30, 46].

Fusarium head blight (FHB), caused mainly by the fungus *Fusarium graminearum* Schwabe, is a destructive disease of wheat and poses a serious threat to the health of consumers of wheat products [2, 49]. Genetic studies in wheat have identified more than 200 useful loci for improvement of complex traits, such as FHB. Unfortunately, many of them remain unused or under-utilized in plant breeding programs mainly because of the complex nature of resistance [6, 7]. Extensive efforts have been made to utilize host resistance for managing this disease. At present, the most effective and widely used QTL for FHB resistance is located on chromosome 3BS of the Chinese wheat varieties Sumai 3 and Wangshuibai [1, 34, 70], which have been further investigated and several additional QTL enhancing the resistance were mapped [43]. Among the QTL identified for resistance to FHB, only seven have been formally designated, i.e., *Fhb1* derived from Sumai 3 [15], *Fhb2* from Sumai 3 [14], *Fhb3* from *Leymus racemosus* [51], *Fhb4* from Wangshuibai [64], *Fhb5* from Wangshuibai and Sumai 3 [65], *Fhb6* from *Elymus tsukushiensis* [8], and *Fhb7* from *Thinopyrum ponticum* [27]. Recent investigations of several other wild relatives of wheat, such as diploid wheatgrass *Leymus racemosus*, *Th. intermedium*, and tetraploid wheatgrass *Th. junceiforme*, have been shown to be highly resistant to FHB [5, 11, 16, 37]. Additionally, some wheat-wild species, including accessions of the St genome of *Th. intermedium* and the E genome of *Th. elongatum*, have been shown highly resistant to FHB, making these species the most successful examples for introgression of elite genes from wild relatives for wheat improvement [32, 54, 67]. Using sequence information obtained from the cloned gene, Rawat et al. [52] studied the origin of *Fhb1* in wheat and related species, and sequenced *Fhb1* gene from a large set of diploid A- genome, S- genome and D- genome accessions. Using sequence data from *Th. elongatum*, Wang et al. [62] mapped *Fhb7* and located it to a 245-kb genomic region. Thus, *Fhb7* resistance differs from *Fhb1* resistance, which depends on a reduction of pathogen growth in spikes, although both confer durable resistance.

Wheat-*Thinopyrum* partial amphiploids play an important role in the transfer of disease-resistant genes from wheatgrass species into common wheat [39, 41]. To date, a number of wheat-*Th. intermedium* partial amphiploids have been developed, such as Zhong 1 to Zhong 5 [56], Otrastsyuskaya (OT), TE-3, TAI8335 [21, 28, 66], TE253 and TE257 [4]. The present study focused on the development of five wheat-*Th. intermedium* partial amphiploids by crossing octoploid tritelytrigia (2n = 8× = 56, AABBDDEE) with *Th. intermedium* and characterized their FHB resistance and genomic constitutions by means of genomic in situ hybridization (GISH) and multicolor fluorescence in situ hybridization (mcFISH).

## Results

### GISH and mcFISH analysis

Chromosome counts indicated that lines HS2-2, HS2-4, and HS2-5 had 56 chromosomes, and lines HS2-14 and HS2-16 had 54 chromosomes (Table 1). GISH analysis using *Th. intermedium* genomic DNA as a probe and common wheat genomic DNA as a blocker revealed that lines HS2-2, HS2-4, and HS2-5 had 14 *Th. intermedium* chromosomes and 42 wheat chromosomes. Lines HS2-14 and HS2-16 had 12 *Th. intermedium* chromosomes and 42 wheat chromosomes. In order to further distinguish the composition of them, both J-genomic DNA from *Th. bessarabicum* and multiplex oligo probes were used with common wheat Chinese Spring as a blocker in the in situ hybridization analysis. Based on the signal patterns and signal density produced by each oligo, A and D genome signals are red or green dot at the arm and terminal of chromosome, B genome centromere usually appears a lot of green dot, so we can distinguish wheat genomes [20, 61].

**Table 1:** Chromosome compositions of the wheat-*Th. intermedium* lines

Line	Pedigree	2n =	No. of wheat chromosomes	No. of *Th. intermedium* chromosomes	
				J genome	St or J^s^ genome
HS2-2	Ganmai8/*Th. intermedium*	56	42	10	4
HS2-4	Ganmai8/*Th. intermedium*	56	42	10	4
HS2-5	Ganmai8/*Th. intermedium*	56	42	14	0
HS2-14	Ganmai8/*Th. intermedium*	54	42	12	0
HS2-16	Ganmai8/*Th. intermedium*	54	42	12	0

**Fig. 1:**
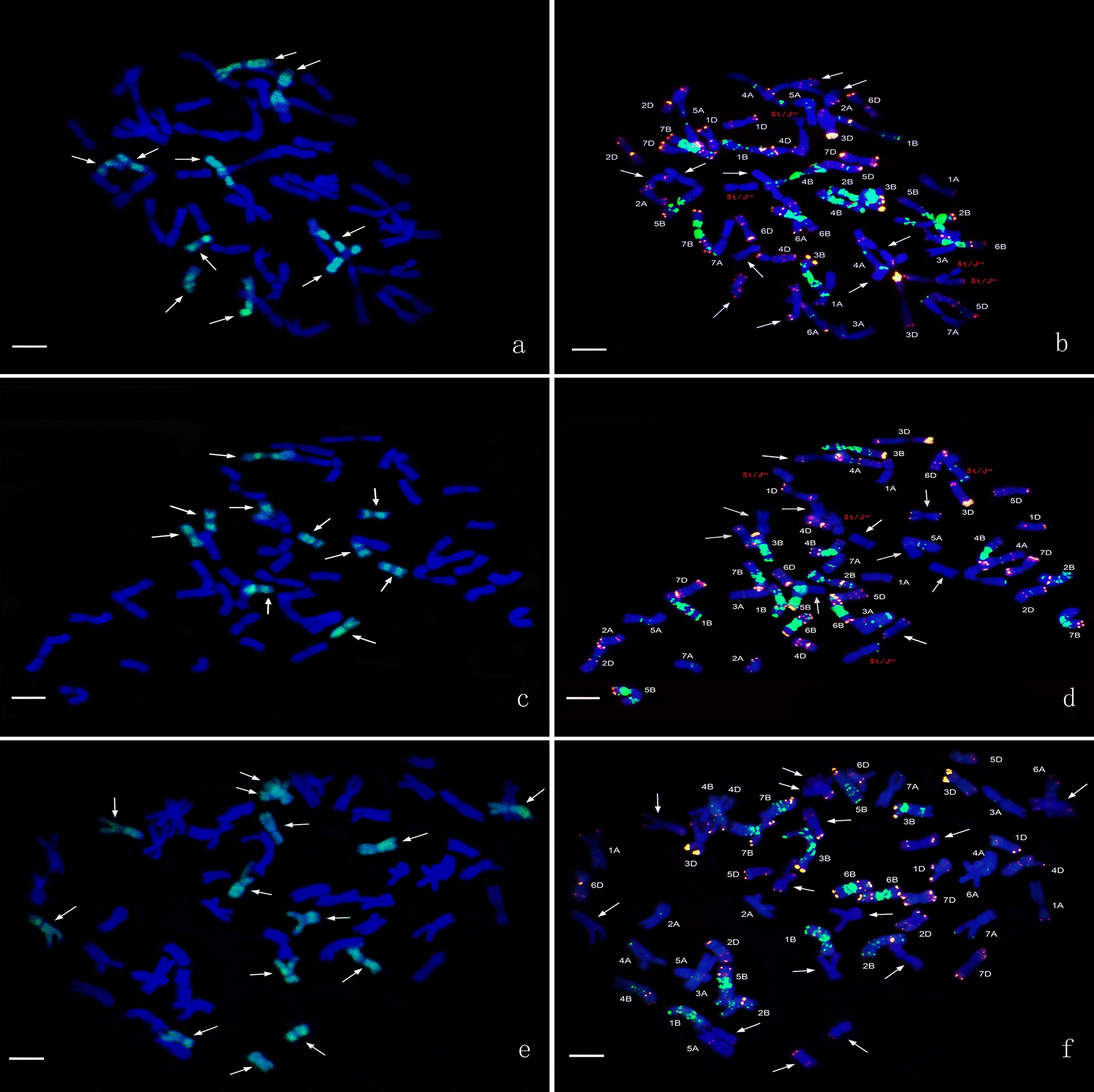
GISH and mcFISH patterns from lines HS2-2, HS2-4 and HS2-5. **a**, **c**, **e** *Th. bessarabicum* (J) genomic DNA labeled with fluorescein-12-dUTP was used as a probe for green signals, and common wheat cultivar Chinese Spring as a blocker. **b**, **d**, **f** The synthetic oligo *pAs1-1*, *pAs1-3*, and *AFA-4* were 5′ end-labelled with 6-carboxytetramethyl-rhodamine (TAMRA) for red signals. The synthetic oligo *pSc119.2-1* and (*GAA*) 10 were 5′ end-labelled with 6-carboxyfluorescein (6-FAM) for green signals. White arrows show the J-genome chromosomes. Bar = 10 μm. **a**, **b** line HS2-2, **c**, **d**: line HS2-4, **e**, **f** line HS2-5

**Fig. 2:**
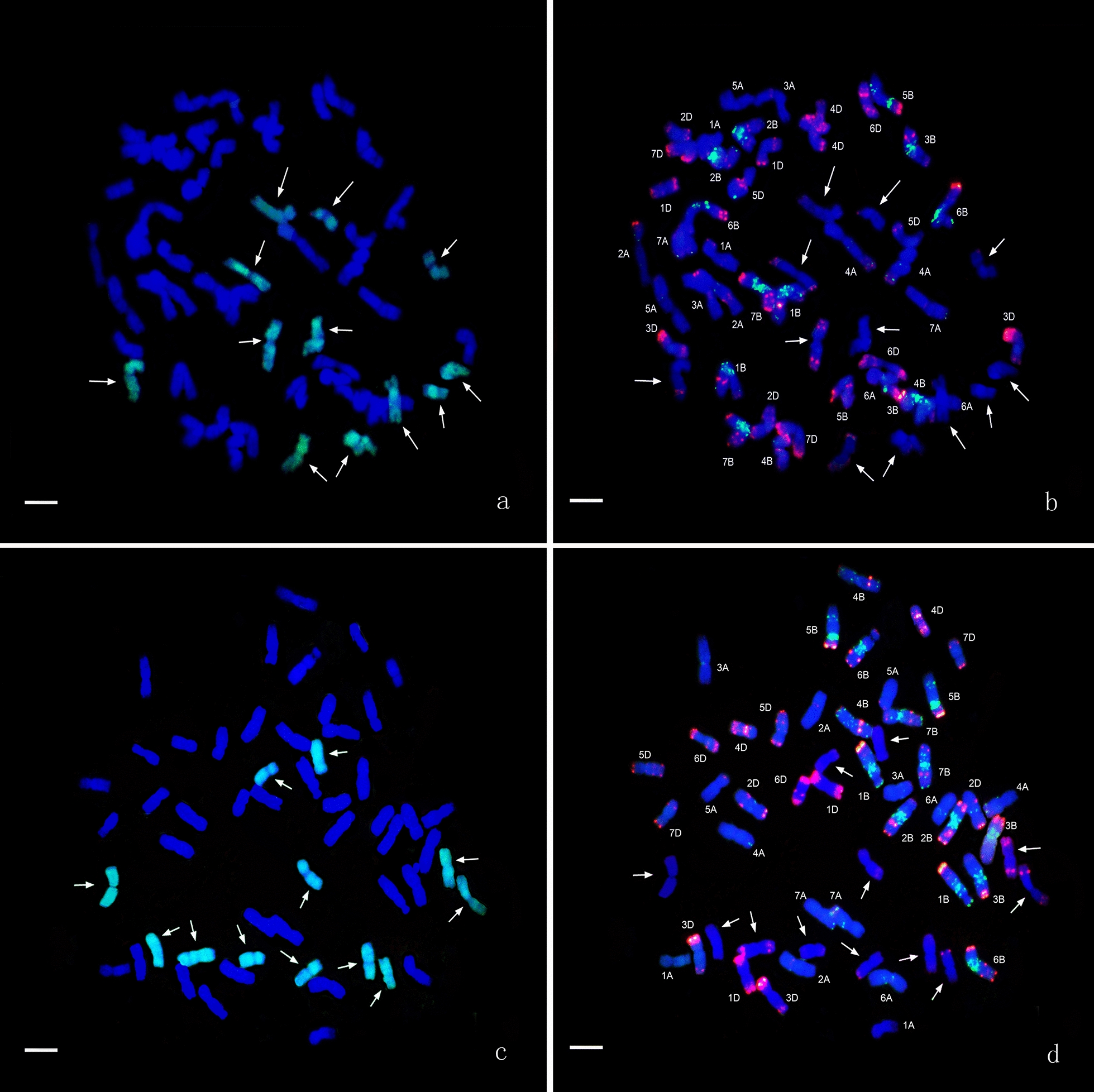
GISH and mcFISH patterns from lines HS2-14 and HS2-16. **a, c**: *Th. bessarabicum* (J) genomic DNA labeled with fluorescein-12-dUTP was used as a probe for green signals, and common wheat cultivar Chinese Spring as a blocker. **b**, **d**: The synthetic oligo *pAs1-1*, *pAs1-3*, and *AFA-4* were 5′ end-labelled with 6-carboxytetramethyl-rhodamine (TAMRA) for red signals. The synthetic oligo *pSc119.2-1* and (*GAA*) 10 were 5′ end-labelled with 6-carboxyfluorescein (6-FAM) for green signals. White arrows show the J-genome chromosomes. Bar = 10 μm. **a**, **b**: line HS 2-14, **c**, **d**: line HS 2-16

GISH analysis of line HS2-2 revealed that 10 chromosomes from *Th. intermedium* displayed a light-green fluorescence signals over most of their lengths except for the terminal and centromeric regions with the J-genome signals (Fig. 1a), and four chromosomes with no signals of J-genome and oligo-probes were St- or J^S^-genome chromosomes from *Th. intermedium* (Fig. 1b). Line HS2-4 with the similar probe pattern of HS2-2 also had 10 chromosomes from *Th. intermedium* with light-green fluorescence signals as the J-genome (Fig. 1c), and four chromosomes with no signals of J-genome and oligo-probes were St- or J^S^-genome chromosomes from *Th. intermedium* (Fig. 1d). Line HS2-5 had 14 J chromosomes from *Th. intermedium* with the light-green J-genome signals (Fig. 1e). Line HS2-14 and HS2-16 had 12 chromosomes from *Th. intermedium* and some chromosomes with the whole light-green J-genome signals (Fig. 2a, c). Analysis of mcFISH with the oligo-probes demonstrated that all the lines maintained the complete set of wheat chromosomes from the A, B, and D genomes (Figs. 1b, d, f, 2b, d).

The above results revealed that line HS2-2 and HS2-4 with 2n = 8× = 56 had 4 chromosomes of St- or J^S^-genome chromosomes and 10 J-genome chromosomes from *Th. intermedium* and 42 wheat chromosomes. The genome composition of line HS2-5 was 42 wheat chromosomes plus 14 J-genome chromosomes from *Th. intermedium*. Lines HS2-14 and HS2-16 had 12 J-genome chromosomes from *Th. intermedium* and 42 wheat chromosomes. Therefore, these lines are identified as the wheat*-Th. intermedium* partial amphiploids.

### Morphological characteristics and seed protein contents

**Fig. 3:**
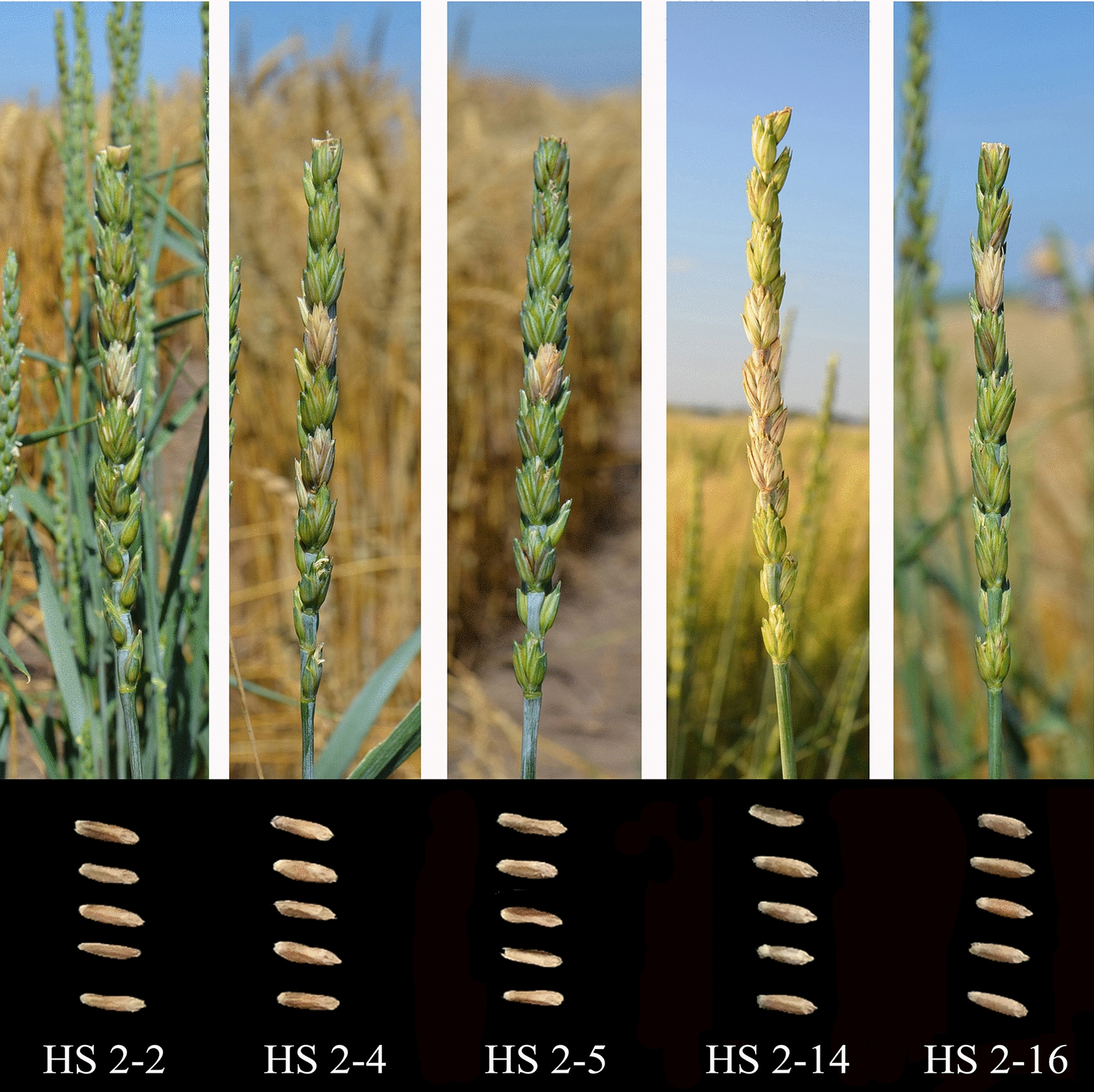
Evaluation of Fusarium head blight resistance and seed morphologies of five wheat-*Th. intermedium* partial amphiploids. The above is the FHB test results in the field (photo taken in July 2017). The below is harvested seeds (photo taken in August 2017). From left to right, lines HS2-2, HS2-4, HS2-5, HS2-14, and HS2-16

In 2016 and 2017, results of morphological characteristics in the field are shown in Table 2. The average plant heights of lines HS2-2, HS2-4, and HS2-5 were lower than both parents, Ganmai 8 and *Th. intermedium*, but those of lines HS2-14 and HS2-16 were similar to their parents. Spike lengths of five lines were similar to the parent Ganmai 8. The number of tillers of the five lines was significantly less than that of the parent *Th. intermedium*, but similar to Ganmai 8 except for line HS2-14. Spikelet numbers per spike of the five lines were similar to the parent of Ganmai 8. Seeds of the main spikes from lines HS2-2 and HS2-4 were not significantly different from Ganmai 8, but higher than *Th. intermedium*. All lines had lower 1,000-kernal weight than Ganmai 8, but higher than *Th. intermedium*. Seed color of the five lines was red (Fig. 3). All the lines were resistant to lodging. Maturity of line HS2-14 and HS2-16 was similar to the local spring wheat cultivar Longmai 33. However, maturity of lines HS2-2, HS2-4, and HS2-5 was later than that of Longmai 33. Seed protein content of the five lines (18.3%-22.5%) was higher than that of the parent Ganmai 8 (18.1%), but lower than *Th. intermedium* (26.0%)*.* Lines HS2-2, HS2-4, HS2-5, and HS2-14 had more than 19% protein content and lines HS2-2 and HS2-5 had the highest values of up to 22.5% and 20.7%, respectively (Table 2).

**Table 2:** The morphological characters for the five wheat-*Th. intermedium* lines, contrals and their parents in 2017

Line	Plant height (cm)	Spike length (cm)	Tiller	Spikelet number	Seeds of main spike	1000-kernal weigh (g)	Seed protein content (%)	Maturity
Longmai33	95^a^	11.2^ef^	4^b^	19^a^	46^b^	41^ab^	17.3^c^	92^bc^
Sumai3	82^b^	8.5^f^	5^b^	16^a^	48^a^	34.5^b^	15.2^c^	80^c^
Ganmai 8	97^a^	13.8^c^	4^b^	18^a^	46^b^	36.3^b^	18.1^c^	95^b^
*Th. intermedium*	82^b^	27.4^ab^	65^ab^	20^a^	25^d^	8.2^c^	26.0^ab^	110^ab^
HS2-2	75^b^	14.5^bc^	4^b^	18^a^	46^b^	30.4^b^	22.5^b^	112^a^
HS2-4	78^b^	13.5^c^	3^b^	18^a^	44^bc^	31.4^b^	20.0^b^	112^a^
HS2-5	80^b^	13.2^c^	3^b^	17^a^	29^ cd^	29.2^bc^	20.7^b^	112^a^
HS2-14	92^ab^	12.7^de^	7^b^	15^a^	35^c^	32.2^b^	19.6^b^	95^b^
HS2-16	93^a^	13.1^ cd^	5^b^	14^a^	32^c^	33.3^b^	18.3^bc^	95^b^

All indices are described by mean; means in a column followed by the same letter(s) are not significantly different at a 5% probability level

### FHB resistance evaluation

In 2016 and 2017, the five partial amphiploids were tested for their FHB resistance in the field. In resistant plants, the fungal infection was restricted to the central inoculated spikelets and did not spread up or down across the spikes (Fig. 3). The field evaluation performed in both 2016 and 2017 showed that all the five lines exhibited better resistance to FHB than the susceptible control Longmai 33 and the resistant control Sumai 3. Values of PSS for lines HS 2-2, HS2-4, HS2-5, and HS2-16 were less than 20% (Table 3). Line HS2-5 showed the best resistance, with the PSS values of 11% and 17% in both 2016 and 2017. Line HS2-2 had a better FHB resistance with the PSS values of 15% and 18% during 2016 and 2017. The values of PSS for lines 2-4 and HS2-16 were 18% and 20%, 16% and 19% in the two years, respectively. In contrast, line HS2-14 had the PSS values of 30% and 28% during 2016 and 2017 (Table 3).

**Table 3:** The evaluation of Fusarium head blight resistance in 2016 and 2017

Line	2016		2017	
	N	PSS	N	PSS
Longmai 33 (S)	30	0.82 ± 0.02^b^	30	0.85 ± 0.02^b^
Sumai 3(R)	30	0.30 ± 0.01 ^b^	30	0.31 ± 0.02 ^b^
HS2-2	30	0.15 ± 0.02^b^	30	0.18 ± 0.03^b^
HS2-4	30	0.18 ± 0.02^b^	30	0.20 ± 0.02^ab^
HS2-5	30	0.11 ± 0.01^b^	30	0.17 ± 0.04^b^
HS2-14	30	0.30 ± 0.03^a^	30	0.28 ± 0.03^a^
HS2-16	30	0.16 ± 0.03^b^	30	0.19 ± 0.03^b^

All indices are described by mean ± standard error

Means in a column followed by the same letters are not significantly different at *P* < 0.05

*N* number of plant spikes, *PSS* the percent of symptomatic spikelets

## Discussion

The production of partial amphiploids is a crucial intermediate step in transferring desirable genes from wheatgrass to wheat [22, 35]. Several wheat-*Th. intermedium* partial amphiploids have been developed and widely exploited as sources of disease resistance in wheat improvement [3, 4, 57, 68]. Georgieva et al. [25] obtained two intergeneric wheat/wheatgrass amphiploid lines H95 and 55(1-57) with high protein content and resistance to certain fungal diseases. Breeding for FHB resistance is difficult because it is a complex trait controlled by multiple genes and influenced by environments [9, 26, 36, 52, 53]. Jauhar and Peterson [33] attempted to transfer FHB resistance of diploid *Th. elongatum* into durum wheat. After screening a series of wheat-*Th. elongatum* derived lines for FHB resistance, Fu et al. [24] indicated that the short arm of chromosome 7E of *Th. elongatum* conferred a high level of resistance to the spread of FHB. The resistance genes from *Th. ponticum* can be pyramided with the currently identified resistance genes in wheat to enhance the genetic diversity and provide more durable resistance of wheat to FHB. Liu et al. [47] reported three common wheat-*Th. ponticum* derived cultivars Xinong 509, Xinong 511 and Xinong 529 with good FHB resistance, which carry a chromosome 7E segment of the decaploid *Th. ponticum*. Four FHB resistant wheat lines L658, L693, L696, and L699 were developed [48], which demonstrates the possibility of using *Th. intermedium* to develop novel wheat germplasm with different disease resistance.

In this study, five wheat-*Th. intermedium* partial amphiploids derived from the octoploid tritelytrigia (2n = 8× = 56, AABBDDEE) and *Th. intermedium* showed the resistance to FHB. GISH and mcFISH results revealed that these lines had 10-14 J-genome chromosomes from *Th. intermedium*. Line HS2-5 had the best resistance to FHB, which indicated that certain J-genome chromosomes of *Th. intermedium* harbored genes for FHB resistance. Chen et al. [12] reported that *T. durum* × *Th. distichum* hybrid lines, AFR4 and AFR5, expressed a significantly higher level of resistance to the spread of FHB compared to the other durum wheat-alien hybrid lines. GISH analysis revealed that all of the alien chromosomes present in lines AFR4 and AFR5 belong to the J-genome. Therefore, *Th. bessarabicum* and *Th. intermedium* as the donor for J-genome chromosomes can be used for breeding FHB resistance. In addition, lines HS2-2 and HS2-5 had not only the least PSS value, but also higher seed protein content (22.5% and 20.7%), indicating that these wheat-*Th. intermedium* amphiploids are considered both as a “breeding bridge” in the transfer of genes from the intermedium wheatgrass to common wheat.

*Th. intermedium*, as an allohexaploid species, is proposed to be formed by an ancient hybridization event between the diploid *Pseudoroegneria strigosa* (2n = 2× = 14, StSt) and a segmental tetraploid carrying J^r^ and J^vs^ genomes [13]. The J^r^ and J^vs^ genomes represent ancestral genomes of the present J^b^ genome of *Th. bessarabicum* and the J^e^ genome of *Th. elongatum* respectively [58]. The J^vs^ genome is distinct from the J^b^ genome as it retains the repetitive sequences from the V genome of *Dasypyrum villosum* L. Candagy [13]. These genomes provide abundant genetic resources for their hybrid progenies.

Identification and tracking of these chromosomes is a prerequisite for directed chromosome engineering. The technologies of GISH and FISH can be used to differentiate and localize *Th. intermedium*, *Th. ponticum*, and *Th. elongatum* chromosomes in wheat backgrounds [63]. In this study, the multiplex oligos containing probe combined with the J-genome DNA as probe were used to discriminate chromosomes from *Th. intermedium* in wheat backgrounds. The J-genome chromosome signals of the five partial amphiploids lines showed two types of hybrid signals. One displayed a light-green fluorescence signals over most of their lengths except for the terminal and centromeric regions as detected by the J-genome probe, such as lines HS2-2, HS2-4 and HS2-5 (Fig. 1a, c, e). This type of hybrid signals was identified by Cseh et al. [13] with the genomic DNA as the probes from the diploid *Ps. strigosa* (St) and *Th. bessarabicum* (J). Another type of signals covered the entire chromosome with a whole light-green fluorescence signal, such as part of J-genome chromosomes of line HS2-16 (Fig. 2c). Two types of hybridization signal on the J-genome chromosomes indicated that among the five partial amphiploid lines developed, the characteristics of these J-genome chromosomes from *Th. intermedium* were not exactly the same as *Th. bessarabicum*. In addition, compared with the conventional FISH probe, synthesized oligonucleotide probe has the advantages of low cost, high sensitivity and high resolution [20]. In this study, although oligo probes were used to distinguish wheat genome, there were some specific signals on J-genome chromosome (Figs. 1, 2), which indicated that the further development of oligo probes on J-genome chromosome would help to improve the recognition efficiency of J-genome chromosome.

## Conclusions

Five wheat-*Th. intermedium* partial amphiploids with FHB resistance and good protein contents were developed by crossing octoploid tritelytrigia (2n = 8× = 56, AABBDDEE) with *Th. intermedium*. Their genomic constitutions was examined by means of GISH and multicolor-FISH. These wheat-*Th. intermedium* amphiploids with the J-genome chromosomes from *Th. intermedium* were identified and can be considered as a valuable source of FHB resistance in wheat breeding.

## Methods

### Plant materials

The plant materials used in this study included *Th. intermedium*, *Th. bessarabicum*, five lines of partial amphiploids and common wheat, Chinese Spring (CS). *Th. intermedium* (2n = 6× = 42 StStJJJ^S^J^S^), Longmai 33 (2n = 6× = 42 AABBDD) and Sumai 3 (2n = 6× = 42 AABBDD) were obtained from the Heilongjiang Academy of Agricultural Sciences, Harbin, China. *Thinopyrum bessarabicum* (2*n* = 2× = 14 JJ or J^b^J^b^) was kindly supplied by Dr. Zengjun Qi, Nanjing Agricultural University, Nanjing, Jiangsu province, China. The lines of wheat-*Th. intermedium* partial amphiploids, HS2-2, HS2-4, HS2-5, HS2-14, and HS2-16, were developed from crosses between Ganmai 8 and *Th. intermedium* at the College of Life Science and Technology of Harbin Normal University, Harbin, Heilongjiang province, China. Ganmai 8 is an octoploid tritelytrigia partial amphiploids (AABBDDEE, 2n = 8× = 56) that were developed from the cross between common wheat line 91C-9 and the partial amphiploid line Yuan 16-3 (2n = 8x = 56, AABBDDEE) in Shanxi Academy of Agricultural Sciences, Taiyuan, Shanxi province, China*.* Chinese Spring wheat (2n = 6× = 42, AABBDD) were provided by the College of Life Science and Technology of Harbin Normal University.

### FHB assessments

The experiment was conducted over two years in two fields: Minzhu field at Crop Resources Institute, Hongjiang Academy of Agriculture Sciences (126º27′E and 51º16.12′N), Harbin, China, in 2016 and 2017; field at Harbin Normal University (126º57′E and 45º87′N), China, in 2016 and 2017. We used a randomized complete block to design an experiment with three replications. The plot consisted of 2.0-m long rows, and the space between rows was 0.4 m. To determine FHB resistance, the field spikelet-cutting method was used to inoculate. Ten microliter of a macroconidial suspension (5000 macroconidia per ml) of the spore-derived isolate of *F. graminearum* No. 4 (provided by Plant Protection Institute, Honglongjiang Academy of Agriculture Sciences, Harbin, China) was injected into 10 random selected plants spikes at early anthesis each. The inoculated spikes were covered with plastic bags for 3 d to maintain the relative humidity and temperature. The data was recorded at 21 d after inoculation. The number of symptomatic spikelets and the total number of spikelets of every tagged spike were counted for the percent of symptomatic spikelets (PSS). Longmai 33 served as the susceptible controls and Sumai 3 served as the resistant control in both fields.

### Morphological observation and protein content of measurement

During 2016 and 2017, each lines was grown in plots consisting of 2.0-m long rows, and spaced 0.4 m that were arranged in a randomized complete block design with three replicates in two fields. Thirty seeds were sown in a row in early April and harvested at end of July. The agronomic performances were investigated in two consecutive years in two fields. From each line, plant height (cm), spike length (cm), number of spikelets per spike, 1000-kernal weigh, awn, and maturity date were recorded in ten randomly sampled individuals from each plot during the growing seasons. Morphological observations were carried out as described previously [42]. Plant height was determined from the ground level to the top of the spike, and spike length was measured from the base of rachis to the top of the spike. In addition, number of spikelets per spike was enumerated and the spikes were threshed in a bench micro-thresher to determine thousand-kernel weight (g). One hundred seeds of each line with the same seed shape were placed into the sample cup. DA7200 Multifunctional Near Infrared Spectromete (Perten, Switzerland) was used for testing the protein content of seed and recorded the data. Simplicity software for data analysis.

### Chromosome preparation

Seeds were germinated at 23.5 °C for 24 h in moist filter paper in Petri dishes, incubated at 4 °C for 48 h, and then returned to 23.5 °C for 27.5 h. Root tips were treated with ice water at 0–4 °C for 24 h, fixed in Carnoy’s fixative (Anhydrous alcohol: Acetic acid = 3:1) for 24 h, and stained in 1% acetic carmine for at least 5 h. Root tips were squashed in 45% acetic acid and observed under a light microscope (BH-2, Olympus, Tokyo, Japan).

### In situ hybridization

Genomic DNA was isolated using a CTAB method [19] from young leaves of the three putative diploid progenitors *Th. bessarabicum* and labeled with fluorescein-12-dUTP by the nick translation method to be used as the probes. Sheared genomic DNA from Chinese Spring (AABBDD, 2n = 42) was used as the blocking DNA. The protocols of GISH and multicolor FISH using the synthesized probes were described by Wang et al. [61]. An oligonucleotide (oligo hereafter) multiplex containing oligos *pAs1-1*, *pAs1-3*, *AFA-4*, (*GAA*) 10, and *pSc119.2*-*1*, was used for identifying A, B and D genomes of wheat that was previously described by Wang et al. [61] and Du et al. [20]. The oligo probes were synthesized by TsingKe biotechnology Co. Ltd. (Beijing, China). The synthetic oligo *pAs1-1*, *pAs1-3*, and *AFA-4* were 5ʹ end-labelled with 6-carboxytetramethyl-rhodamine (TAMRA) for red signals. The synthetic oligo *pSc119.2-1* and (*GAA*) 10 were 5ʹ end-labelled with 6-carboxyfluorescein (6-FAM) for green signals. Hybridization stringency (%) = 100 + hybridization temperature (T_h_)- melting temperature (T_m_) = 100 + 37 °C (T_h_)—105 °C (T_m_) = 32%. Photographs were captured with a Leica DM6000B fluorescence microscope (Leica, Mannheim, Germany) equipped with a digital camera (Leica model DFC480).

### Statistical analysis

Significant differences in the means of different genotypes for PSS were determined by the multiple samples *t*-test at *P* < 0.05 using IBM SPSS Statistics 19 software (SPSS Inc., Chicago, IL), and the significance of differences in the same genotype indices between inoculated and non-inoculated plants was also determined by the independent samples *t*-test at *P* < 0.05 with the same software.

## Data Availability

All data generated or analyzed during this study are included in this published article.

## Abbreviations

GISH: genomic in situ hybridization
FISH: fluorescence in situ hybridization
mcFISH: multicolor fluorescence in situ hybridization
PSS: percent of symptomatic spikelets
FHB: fusarium head blight
